# Learning pharmacometric covariate model structures with symbolic regression networks

**DOI:** 10.1007/s10928-023-09887-3

**Published:** 2023-10-21

**Authors:** Ylva Wahlquist, Jesper Sundell, Kristian Soltesz

**Affiliations:** Department of Automatic Control, Lund University, P.O. Box 118, 221 00 Lund, Sweden

**Keywords:** Pharmacometrics, Covariate modeling, Pharmacokinetics, Symbolic regression, Neural networks

## Abstract

Efficiently finding covariate model structures that minimize the need for random effects to describe pharmacological data is challenging. The standard approach focuses on identification of relevant covariates, and present methodology lacks tools for automatic identification of covariate model structures. Although neural networks could potentially be used to approximate covariate-parameter relationships, such approximations are not human-readable and come at the risk of poor generalizability due to high model complexity.In the present study, a novel methodology for the simultaneous selection of covariate model structure and optimization of its parameters is proposed. It is based on symbolic regression, posed as an optimization problem with a smooth loss function. This enables training of the model through back-propagation using efficient gradient computations.Feasibility and effectiveness are demonstrated by application to a clinical pharmacokinetic data set for propofol, containing infusion and blood sample time series from 1031 individuals. The resulting model is compared to a published state-of-the-art model for the same data set. Our methodology finds a covariate model structure and corresponding parameter values with a slightly better fit, while relying on notably fewer covariates than the state-of-the-art model. Unlike contemporary practice, finding the covariate model structure is achieved without an iterative procedure involving manual interactions.

## Introduction

Pharmacokinetics (PK) are the dynamics governing drug uptake, distribution and elimination from the body. For many drugs, it is common to assert a low-order linear and time-invariant (LTI) compartment model for PK modeling. Such low-order models typically capture the uptake, distribution, and elimination dynamics adequately. Increasing model complexity through, for example, additional compartments results in models where the parameters are not practically identifiable from data collected during clinical trials or clinical practice.

While suitable PK model structures (e.g. number of compartments and topology) for common drugs can be established from data, a notable challenge exists in that the parameter values that explain the PK for one individual, are often not suitable for another. Such inter-individual variability is partly explainable by individual-specific features that are referred to as covariates, and partly attributed to random effects.

The purpose of pharmacometric covariate modeling is to identify covariates (i.e., fixed effects) responsible for inter-individual variability, thus minimizing random effects. The gold standard is to approach this problem in a Bayesian setting using mixed-effect modeling, for which there exists both mature [1] and novel [2] tools. However, to apply mixed-effect modeling, one needs to decide which of possibly many covariates (e.g. age, body mass, gender, or genetic factors) to include. One also needs to decide on a parametric function that maps included covariates to parameters of the PK model. There is a tradition of using functions that are of sufficiently low complexity to be human-readable, and some function classes are more popular than others [3, 4]. However, for data sets including numerous covariates, selection of covariates and functions to consider is a combinatorial problem, where the search space may become limiting. Furthermore, due to step-wise approaches typically used for covariate identification, functions including multiple covariates are typically only considered if supported by prior knowledge [5].

Recently, the interest in combining machine learning (ML) and pharmacometrics [6] has increased. For example, ML has shown promising results in concentration predictions [7], identification of influential covariates [8, 9], as well as parameter regression and model selection with the use of genetic algorithms and neural networks (NNs) [10]. The ML methods have been able to match, and even beat, classic NLME modeling at a much higher computational speed. However, using ML to select influential covariates and identify the structural model simultaneously has, to our knowledge, not yet been studied.

In the present paper, we provide an automatic method for simultaneous covariate selection and identification of covariate functions using an adaptation of ML. Similarly to the current standard approach, we maintain simple human-readable expressions. The method is based on symbolic regression [11], that approximates the underlying combinatorial problem with a smooth continuous one, thus enabling the use of efficient gradient-based optimization methods.

To illustrate utility, we apply our methodology to a large data set for the drug propofol and compare the resulting covariate model with the current state-of-the-art model [4]. Our method produces a model with increased predictive performance, using fewer covariates. Furthermore, the covariate model is optimized autonomously in contrast to the prevalent iterative and manual procedure.

## Methods

**Fig. 1:**
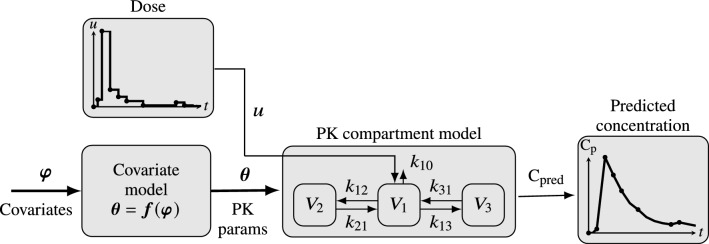
Mammillary three-compartment model example illustrating our novel method. The objective is to automatically learn the covariate model ${\varvec{f}}$ that maps a known covariate vector ${\varvec{\varphi }}$ (comprising e.g., age, gender, or genetic factors) to the parameter vector ${\varvec{\theta }}$ (e.g. rate constant $k_{\cdot \cdot }$ and volumes $V_\cdot$) of a fixed-structure pharmacometric (PK) model. The method is data-driven in that it uses drug administration profiles (time series data) *u*, and model-based in that it assumes a PK model of known structure. In this example, ${\varvec{f}}$ is learned to minimize some error measure between observed (i.e. measured from samples) blood plasma concentrations and corresponding predictions $C_\text {pred}$ by the model. Dots in the graphs show instances of dose changes and blood samples, respectively

The method described in this paper aims to automatically learn closed-form pharmacometric parameter–covariate relationships from data. We will consider learning expressions for PK model parameters from drug administration and resulting blood plasma concentration data, both of which are time series, but not necessarily synchronous or equidistant samples. The overarching setup for this is shown in Fig. 1 and we will focus on a concrete example based on a large multi-study data set published in [4] for the anesthetic drug propofol, commonly modeled with a mammillary three-compartment model [12].

### Data set

We demonstrate our method on a data set composed of propofol plasma concentration observations from 1031 individuals^1^ from 30 clinical studies aggregated by Eleveld et al. [4], from here on referred to as the *data set*. Ethical approvals of the underlying studies are declared in the original publications, referenced by [4]. The data set contains 15,433 observations, of which 11,530 were arterial and 3903 venous. Of the 1031 individuals, there were 670 males and 361 females with ages ranging from 27 weeks to 88 years, and weights ranging from 0.68 to 160 kg, see Table 1.

**Table 1:** Covariate candidates considered in the PK model of [4] as well as in our modeling. Individuals of the data set fall within the reported ranges

Covariate	Interpretation	Unit	Range
$\varphi _1$	Age	Years	0–88
$\varphi _2$	Weight	kg	0.68–160
$\varphi _3$	BMI	kg m${\phantom{0}}^{-2}$	6.2–52.8
$\varphi _4$	Gender	Male/female	670 M/ 361 F
$\varphi _5$	Blood sampling site	Arterial/venous	727 A/ 306 V

The main reason for choosing this data set as a demonstrator is that propofol is a drug with well-studied pharmacokinetics. Evidence of this, which also constitutes a benchmark for our demonstrator, is the model presented in [4]. Furthermore, the data set is that it has been openly disclosed by Eleveld et al., enabling transparent third-party analysis of our work.

In our model development, we consider age, weight, BMI, gender, and blood sampling site (arterial or venous) as potential covariates of our PK model. These are the same candidates as considered in [4], where individual demographics were disclosed as part of the data set.

The data was pre-processed the same way as in [4]: data points corresponding to subsequent infusion changes spaced closer than 1 s apart in the time dimension, or 0.5 $\mu \textrm{gs}^{-1}$ in the dose dimension were merged.

### Pharmacokinetic model

We consider a three-compartment mammillary model to describe the pharmacokinetics of propofol. The drug concentration $x_i$ $[\mu \textrm{gL}^{-1}]$ in compartment $i \in \{1,2,3\}$ is

1a $$\begin{aligned} {\dot{x}}_1&= - (k_{10} + k_{12} + k_{13}) x_1 + k_{21} x_2 + k_{31} x_3 + \frac{1}{V_1} u, \end{aligned}$$

1b $$\begin{aligned} {\dot{x}}_2&= k_{12} x_1 - k_{21} x_2, \end{aligned}$$

1c $$\begin{aligned} {\dot{x}}_3&= k_{13} x_1 - k_{31} x_3, \end{aligned}$$

where $k_{ij}$ describes the drug transfer rate [1/s] from compartment *i* to *j*. The drug is administered at rate *u* [$\mu \textrm{gs}^{-1}$] to the central compartment ($i=1$), which is also where the propofol plasma concentration is measured. The volume of the central compartment is $V_1$ [L].

In the literature, the equivalent parameterization of volumes ($$V_1, V_2, V_3$$) and clearances ($$CL, Q_2, Q_3)$$ constitutes a common alternative to Eq. 1. The conversions between these parameterizations are

2a $$\begin{aligned} CL&= k_{10} V_1\,{[\textrm{Ls}^{-1}]}, \end{aligned}$$

2b $$\begin{aligned} Q_2&= k_{12} V_1\,{[\textrm{Ls}^{-1}]}, \end{aligned}$$

2c $$\begin{aligned} Q_3&= k_{13} V_1\,{[\textrm{Ls}^{-1}]}, \end{aligned}$$

2d $$\begin{aligned} V_2&= \frac{k_{12}}{k_{21}} V_1\,{[L]}, \end{aligned}$$

2e $$\begin{aligned} V_3&= \frac{k_{13}}{k_{31}} V_1\,{[L]}. \end{aligned}$$

We have chosen to implement our method using the parameterization in Eq. 1 due to numeric benefits. These are further explained in [13], where we developed a fast and natively differentiable simulator for the three-order mammillary model.

### Covariate model

The covariate model, shown in Fig. 1, is expressed as a function ${\varvec{f}}$ that maps the covariate vector ${\varvec{\varphi }}=[\varphi _1,\ldots ,\varphi _{n_{\varphi }}]^\top$ to the vector ${\varvec{\theta }}=[\theta _1,\ldots ,\theta _{n_{\theta }}]^\top$ of PK model parameters. Thus ${\varvec{f}}$ has components $f_1,\dots ,f_{n_{\theta }}$, each mapping the covariate vector ${\varvec{\varphi }}$ to one of the $n_\theta$ PK model parameters.

In our example with the three-compartment model for propofol, there are $n_{\varphi } = 5$ covariates and $n_{\theta } = 6$ PK model parameters according to Table 1. To not favor covariates based on their scale, all input covariates were normalized during model development. Continuous inputs (age, weight and BMI) were scaled from 0 to 1, and categorical inputs (gender and blood sampling site) were scaled to $\pm 0.5$.

### Predictive performance

To assess the quality of a particular covariate model candidate ${\varvec{f}}$, we need a performance measure that captures how well ${\varvec{f}}$ reflects the training data. Most optimization methods, including the ones used in this paper, relies on this measure being scalar.

For comparability, we employ the same scalar performance measures as those used in [4]: We train our model to minimize an ensemble average of absolute logarithmic error (ALE). Subsequently, we evaluate predictive performance in terms of ALE, and three additional error measures: logarithmic error (LE), prediction error (PE), and absolute prediction error (APE).

For each individual in the data set, there is a vector of observation–prediction errors, where each entry corresponds to the difference between a blood sample observation and the value predicted by the model. Observation-prediction errors could thus be computed for each sample, over a time series for one individual, or the entire data set. This prompts a consistent notation and we index by *ij* the error over a sample *j* for individual *i*, whereas a total error over a time series for an individual is indexed by *i*.

The per-sample (absolute) logarithmic error (A)LE [14] is thus

3a $$\begin{aligned} \text {LE}_{ij}&= \ln (C_{\text {obs}_{ij}}/C_{\text {pred}_{ij}}), \end{aligned}$$

3b $$\begin{aligned} \text {ALE}_{ij}&= |\text {LE}_{ij} |, \end{aligned}$$

where $C_{\text {obs}_{ij}}$ are observed (measured) plasma concentrations and $C_{\text {pred}_{ij}}$ are corresponding predictions produced by the model.

Similarly, the per-sample (absolute) prediction error (A)PE [15] is

4a $$\begin{aligned} \text {PE}_{ij}&= \left( \dfrac{C_{\text {obs}_{ij}}-C_{\text {pred}_{ij}}}{C_{\text {pred}_{ij}}} \right) \cdot 100 \% \end{aligned}$$

4b $$\begin{aligned} \text {APE}_{ij}&= |\text {PE}_{ij} |. \end{aligned}$$

To avoid divisions by zero if $C_{\text {pred}_{ij}}=0$, a special casing is needed where the error is set to zero for such samples.

Using Eqs. 3 and 4, corresponding per-individual errors were computed by taking the median, resulting in the median absolute logarithmic error (MdALE):

5 $$\begin{aligned} \text {MdALE}_i = \text {median} \left( \text {ALE}_{ij} \right) , \quad j = 1,\dots ,n_i, \end{aligned}$$

where $n_i$ is the number of entries of the time series from individual *i*.

To train the model of [4] and the ones considered here, a single scalar loss function representing model fit is required. This is obtained by averaging across the individuals. Training the covariate model to minimize ALE thus translates into minimizing

6 $$\begin{aligned} J_\text {ALE}=\dfrac{1}{n} \sum _{i=1}^{n} \text {MdALE}_i, \end{aligned}$$

where *n* is the number of individuals in the data set.

Similarly to the analysis performed in [4], ALE and APE were used as indicators of model accuracy and LE and PE were used as indicators of bias. Values closer to zero for ALE and APE reflect better accuracy and values closer to zero for LE and PE indicate less bias.

Clinically acceptable ranges for MdPE${\phantom{0}}_i$ and MdAPE${\phantom{0}}_i$ are $10-20 \%$ and $20-40 \%$, respectively [4, 15, 16]. This translates to acceptable clinical ranges for bias measure MdLE${\phantom{0}}_i < 0.18$ and accuracy measure MdALE${\phantom{0}}_i < 0.34$.

Of note, the choice of loss function will affect how outliers are penalized, where using an average loss across individuals would for example penalize outliers more than a median loss over individuals.

### Symbolic regression networks

**Fig. 2:**
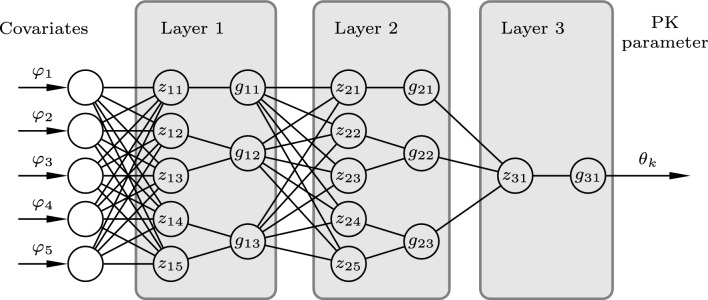
Symbolic regression network with three layers, each marked by a gray box. The output of node $z_{li}$ at layer *l* is the $i ^{th}$ component of ${\varvec{z}}_{l} = W_l {\varvec{x}}_l + {\varvec{b}}_l$, where ${\varvec{x}}_l$, $W_l$ and ${\varvec{b}}_l$ are the input vector, weight matrix, and bias vector of that layer. The base expressions $g_{li}$ acting on $z_{li}$ take on the role of activation functions used in ordinary ANNs. For example, the output of the first layer (and therefore input to the second layer) is ${\varvec{x}}_2 = {\varvec{g}}_1(W_1 {\varvec{x}}_1 + b_1)$. Input and output of the network is the covariate vector ${\varvec{\varphi }}= {\varvec{x}}_1$ and the PK parameter $\theta _k = {\varvec{x}}_4$, respectively

At the core of our methodology lies a small artificial neural network (ANN) with a specific structure, that of a *symbolic regression network* [17], representing a simple closed-form expression. In our case, the purpose is to learn human-readable closed-form expressions describing the covariate model ${\varvec{f}}$. A schematic illustration of such network is shown in Fig. 2.

In general, ANNs can be viewed as flexible function approximators that can be trained to represent input–output mappings that fit available data. An ANN consists of $n_l$ layers, where the output vector ${\varvec{x}}_{l+1}$ of layer *l* is obtained by applying a vector of nonlinear *activation functions* ${\varvec{g}}_l$ to an affine transformation ${\varvec{z}}_l$ to the layer input ${\varvec{x}}_l$:

7a $$\begin{aligned} {\varvec{z}}_l&=W_l{\varvec{x}}_l+{\varvec{b}}_l, \end{aligned}$$

7b $$\begin{aligned} {\varvec{x}}_{l+1}&={\varvec{g}}_l({\varvec{z}}_l). \end{aligned}$$

The linear weight matrices $W_l$ and bias vectors ${\varvec{b}}_l$ constitute the free parameters used to train the network. In the conventional case, the components of the activation functions ${\varvec{g}}_l$ are monotonously increasing functions, such as sigmoids [18]. In contrast, a symbolic regression network can be understood as an ANN with the additional constraint that the resulting approximator ${\varvec{f}}$ should be a human-readable closed-form expression of a mathematical function [17, 19–21]. Training of symbolic regression networks is therefore often referred to as equation learning [17]. The methodology has gained broad attention for its ability to produce impressive results in discovering (known) physical laws from data [22].

The activation functions ${\varvec{g}}$ of a symbolic regression network represent mathematical functions that we refer to as *base expressions*. In standard symbolic regression, a sequence of topologies with different base expressions and scalar parameters–of which the weight matrices and bias vectors in Eq. 7 would constitute a special case–are evaluated in search of one that fits the available data well [23]. Conventional equation learning is thus a combinatorial problem, with poor (exponential) time complexity, and therefore often approached using genetic algorithms [23].

Instead of relying on random perturbations as in genetic algorithms, we use gradient-based optimization methods common to conventional ANN training. To ensure human-readability, we enforce sparsity of the ANN by alternating training epochs with pruning epochs, in which the least important parameters are removed from the network. We next rely on a concrete example based on the Eleveld data set, to describe and illustrate this approach.

We set out with a nominal (unpruned) network of Fig. 2. It constitutes our nominal representation of $f_k$, where the inputs are the covariates according to Table 1. Thus, the output of the network represents one of the PK parameters $\theta _k$, such as $k_{10}$ or $V_1$ of Eq. 1. For a model with $n_\theta$ PK parameters, we use a parallel interconnection of $n_\theta$ symbolic regression networks, each modeling one component $f_k$ of ${\varvec{f}}$, mapping the covariate vector ${\varvec{\varphi }}$ to each PK parameter $\theta _k,\ k=1,\dots ,n_\theta$.

In our example, the base expressions of each layer $l \in \{1,2,3 \}$, with input vector $$\varvec{z}_l = \begin{bmatrix} z_{l1},&z_{l2},&\dots \end{bmatrix} ^\top$$, were chosen to cover previously published PK models for propofol, such as [4] and [12]:

$$\begin{aligned} \varvec{g}_1(\varvec{z}_1)&= \begin{bmatrix} z_{11}\\ z_{12} \cdot z_{13}\\ |z_{14} |^{z_{15}}, \end{bmatrix}\\ \varvec{g}_2(\varvec{z}_2)&= \begin{bmatrix} z_{21}\\ z_{22} \cdot z_{23} \\ \frac{z_{24}}{z_{25} + 1}, \end{bmatrix}\\ g_3(z_3)&= |z_{3} |, \end{aligned}$$

where the $g_3$ assures positive output of the final layer. The division in $\varvec{g}_2$ has the term one in the denominator to assure that the output does not blow if $z_{25} \ge 0$ approaches zero.

#### Training

Minimizing the *loss function* Eq. 6 across trainable parameters of the covariate model ${\varvec{f}}$ is referred to as training. In our case, these parameters are stacked into a vector ${\varvec{\gamma }}$, made up by the elements of all weight matrices and bias vectors. Since the data set is static, we can view training as minimization of the scalar-valued function $J_\text {ALE}({\varvec{\gamma }})$. In order to do so, we need to evaluate $J_{ALE}({\varvec{\gamma }})$. Doing so requires simulation of one PK model for each data set individual, to obtain the predicted plasma concentration at each observation time instance. This can be efficiently done using the method introduced in [13].

In addition to the supporting fast simulation, the method of [13] enables exact and efficient evaluation of derivatives of individual prediction errors with respect to the trainable model parameters. This allows us to train the covariate model ${\varvec{f}}$ using conventional ANN back-propagation, using the stochastic gradient-based optimization algorithm ADAM [24]. As with artificial neural networks in general, establishing formal convergence guarantees is challenging. However, in practice both standard deep learning networks and our symbolic regression do converge to models that fit data adequately well. Similarly to the deep learning case, convergence rate will also vary with data, choice of activation functions, learning rate, and initialization of the training. Our implementation, using the neural network package Flux [25], relies on the differential programming capabilities of the Julia language [26]. A full disclosure of our implementation is found in the GitHub repository [27].

#### Pruning

To obtain simple human-readable expressions from an initially dense symbolic regression network, such as the one in Fig. 2, we alternate between parameter training of the fixed network structure and pruning of the network.

**Fig. 3:**
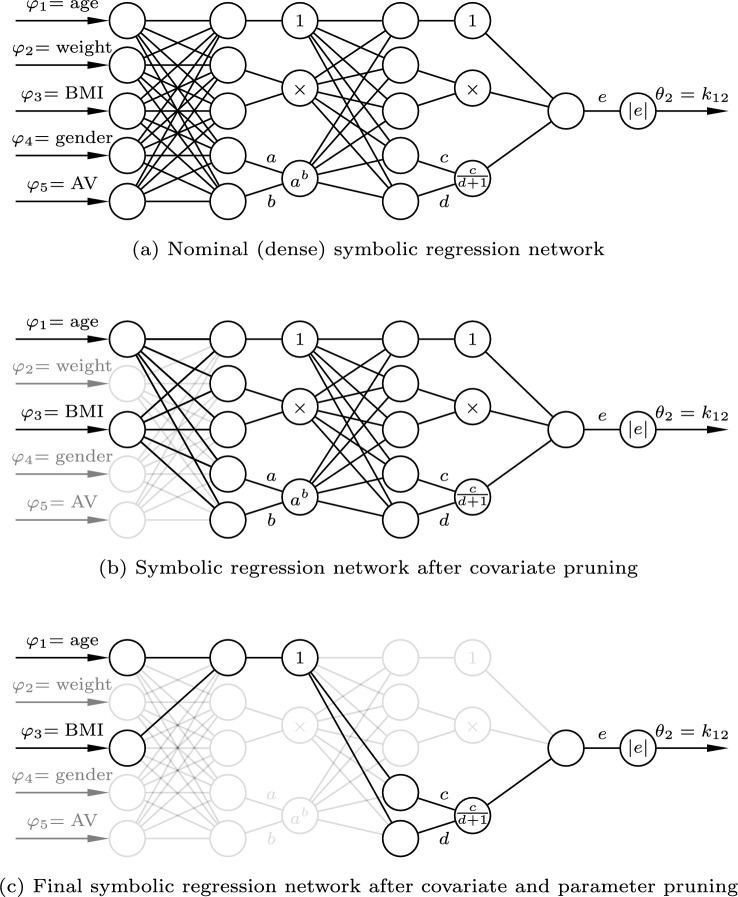
Pruning sequence of a symbolic regression network with output pharmacokinetic parameter $\theta _2 = k_{12}$. Input covariates are age, weight, gender, and arterial or venous sampling (AV). The nominal network has three dense layers, each followed by base expressions such as 1 (feedforward), multiplication, power function, division and absolute value. A black line represents a connection between two nodes, and a gray line represents a pruned (removed) connection. The final network represents the covariate expression of Eq. 10b

The goal of this pruning is to obtain a sparse network structure, which translates into a readable expression of the corresponding covariate model. In the pruning process, we remove covariates and network parameters that have relatively little influence on the network output. This process is visually exemplified in in Fig. 3.

It is desirable to only include as many covariates as needed to explain the data [5]. Therefore, we start by identifying and removing the least important covariates from the symbolic regression network to obtain expressions with fewer covariates. Next, we prune network parameters ${\varvec{\gamma }}$ (linear weights and biases) to obtain simple covariate expressions. Pruning a network parameter is achieved by fixing its value to zero and removing it from the vector ${\varvec{\gamma }}$ of trainable parameters.

Before each pruning iteration, we train the network until convergence. This translates into finding a local minimum of the loss function in Eq. 6. At such minima, the partial derivatives of the loss function with respect to the trainable parameters are zero. Therefore, the second derivatives define the first non-zero terms of the Taylor series expansion that describe local parameter sensitivities, as further explained in Appendix A. The second-order derivatives make up the elements of the Hessian matrix. Specifically its diagonal elements represent sensitivities in the corresponding individual parameters.

In the Taylor series expansion, the second-order terms take on the form

8 $$\begin{aligned} S(\gamma _k) = \gamma _k^2 H_{k}, \end{aligned}$$

where $H_{k}$ is the $k^\text {th}$ Hessian diagonal element of the loss function with respect to the network parameter $\gamma _k$. $S(\gamma _k)$ denotes *salience* of a parameter $\gamma _k$ [28].

For pruning of the network parameters ${\varvec{\gamma }}$, Eq. 8 may be used directly. However, computing the salience for a covariate is not as straightforward since the Hessian elements would differ between individuals. A solution to this is to sum the salience contribution of each individual,

9 $$\begin{aligned} S(\varphi _k) = \sum _{i = 1}^{n} \varphi _{ik}^2 (H_{i})_k, \end{aligned}$$

where $(H_{i})_k$ is the $k^\text {th}$ diagonal element of the Hessian, $H_i$, determining the sensitivity of the covariate $\varphi _{ik}$ for individual *i*, and *n* is the number of individuals.

We use the Zygote package [29] in Julia to compute the Hessian diagonal elements. In Appendix A, we give a more detailed description of the Hessian-based pruning method, providing mathematical insight into this methodology.

#### Recipe: Symbolic regression

A compact summary of our training and pruning scheme is provided below. Initially, we start with a nominal symbolic regression network, like the one shown in Fig. 2. It is sequentially trained and pruned until we obtain a final expression of sufficient complexity and fit. Our training and pruning sequence of a symbolic regression network is as follows:

1. Choose a nominal symbolic regression network architecture, and corresponding base expressions.
2. Train the network until convergence.
3. Compute the salience of each (remaining) covariate, $S(\varphi _{k})$ of Eq. 9.
4. Sort the covariates by saliency and remove the covariate with the smallest salience.
5. If the desired final number of covariates is reached, continue to step 6, otherwise return to step 2.
6. Train the reduced network until convergence.
7. Compute the salience of each (remaining) trainable network parameter, $S(\gamma _k)$ of Eq. 8.
8. Sort the network parameters by saliency and remove *N* parameters with the smallest salience.
9. If the desired final number of network parameters is reached, continue to step 10, otherwise return to step 6.
10. Train the reduced network until convergence.
11. Convert the resulting network to a readable functional expression.

The number of covariates and network parameters to keep in the final symbolic regression network is a trade-off between fit to data and complexity of the final expression. In our example, illustrated in Fig. 3, we remove one covariate at each pruning iteration, until only two covariates are left. Next, we remove the $N=10$ least sensitive parameters in the first parameter pruning iteration, and then one parameter per subsequent pruning iteration until only twelve network parameters are left. More details of the pruning and training can be found in [27]. In [30], we demonstrate that our methodology can identify functions of known shapes correctly.

Initialization of the parameter values associated with step 1 of the recipe affects the fit of the resulting final model. To mitigate the risk of poor model fit due to an unfortunate initialization, the recipe could be run several times, where the best fitting model is kept. In our example, we have executed the recipe eight times.

### Limits of performance

Structural mismatch between the asserted PK model structure Eq. 1 and the data, in combination with measurement errors, induce upper and lower limits on prediction errors of the trained covariate model. Here we explain how these limits can be characterized by training two additional models.

Even with a very complex covariate model, one cannot expect perfect fit to data (zero loss). This is because the fixed (three-compartment) PK model structure is only an approximation of the actual pharmacokinetics, combined with (blood sample) measurement errors. Part of the loss remaining after applying our covariate modeling scheme can thus be attributed to this mismatch. To indicate how much, we optimize one set of (three-compartment) PK parameters for each individual in the data set. This results in a completely covariate-free model. While it will fit data better than any covariate model, it does not generalize. This makes it practically useless for purposes other than providing an upper limit for performance.

Another natural question to ask is how much we gain (in terms of loss) by considering covariate dependencies. To do this, we optimize a constant, i.e. covariate-free, model—one where all individuals share the same (three-compartment) PK parameter values. This model does constitute a lower bound of the achievable predictive performance that any covariate-based model should beat.

For these two additional models, PK parameter optimization was done with the optimization package Optim.jl [31], to minimize the same loss as for our covariate model based on symbolic regression. Implementation details can be found in [27].

## Results

Applying the proposed methodology to the Eleveld data set [4], resulted in the following covariate model, mapping covariates to rate constants and central compartment volume of the PK model in Eq. 1:

10a $$\begin{aligned}&k_{10} = 0.00441 \frac{\text {WGT}}{\text {WGT}_{\text {max}}}+0.00342\,[\textrm{s}^-1] \end{aligned}$$

10b $$\begin{aligned}&k_{12} = \left|\frac{0.158 \frac{\text {AGE}}{\text {AGE}_{\text {max}}}- 0.00431 \frac{\text {BMI}}{\text {BMI}_{\text {max}}}-0.188 }{0.64 \frac{\text {AGE}}{\text {AGE}_{\text {max}}}- 0.0174 \frac{\text {BMI}}{\text {BMI}_{\text {max}}}- 0.743}\,\right|{[\textrm{s}^-1]} \end{aligned}$$

10c $$\begin{aligned}&k_{13, \text {male}} = \frac{0.0058 \left( \frac{\text {AGE}}{\text {AGE}_{\text {max}}}\right) ^2 + 0.00208\frac{\text {AGE}}{\text {AGE}_{\text {max}}}+ 0.0026}{2.75 \left( \frac{\text {AGE}}{\text {AGE}_{\text {max}}}\right) ^2 + 0.985 \frac{\text {AGE}}{\text {AGE}_{\text {max}}}+ 0.601} {[\textrm{s}^-1]} \end{aligned}$$

10d $$\begin{aligned}&k_{13, \text {female}} = \frac{0.0058 \left( \frac{\text {AGE}}{\text {AGE}_{\text {max}}}\right) ^2 - 0.00208\frac{\text {AGE}}{\text {AGE}_{\text {max}}}+ 0.0026}{2.75 \left( \frac{\text {AGE}}{\text {AGE}_{\text {max}}}\right) ^2 - 0.985 \frac{\text {AGE}}{\text {AGE}_{\text {max}}}+ 0.601} {[\textrm{s}^-1]} \end{aligned}$$

10e $$\begin{aligned}&k_{21} = \left|0.00408 \left( \frac{\text {BMI}}{\text {BMI}_{\text {max}}}\right) ^2 - 8.16 \cdot 10^{-4}\frac{\text {BMI}}{\text {BMI}_{\text {max}}}- 0.0057 \frac{\text {BMI}}{\text {BMI}_{\text {max}}}\frac{\text {WGT}}{\text {WGT}_{\text {max}}}+ 0.00218 \right|{[\textrm{s}^-1]} \end{aligned}$$

10f $$\begin{aligned}&k_{31, \text {male}} = 4.52 \cdot 10^{-5} + 1.92 \cdot 10^{-5} \frac{\text {AGE}}{\text {AGE}_{\text {max}}}{[\textrm{s}^-1]} \end{aligned}$$

10g $$\begin{aligned}&k_{31, \text {female}} = 4.52 \cdot 10^{-5} - 1.92 \cdot 10^{-5} \frac{\text {AGE}}{\text {AGE}_{\text {max}}}{[\textrm{s}^-1]} \end{aligned}$$

10h $$\begin{aligned}&V_{1} = 0.0596 \frac{\text {AGE}}{\text {AGE}_{\text {max}}}+ 18.7 \frac{\text {WGT}}{\text {WGT}_{\text {max}}}\nonumber \\&-13.7\left( \frac{\text {WGT}}{\text {WGT}_{\text {max}}}\right) ^2 - 3.5 \frac{\text {AGE}}{\text {AGE}_{\text {max}}}\frac{\text {WGT}}{\text {WGT}_{\text {max}}}- 0.0557 {[L]}. \end{aligned}$$

AGE, WGT, BMI represent age [years], weight [kg] and body mass index $[{\textrm{kg m}}^-{1}]$. The subscript max represent the input normalization where $\text {AGE}_{\text {max}} = 88$ years, $\text {WGT}_{\text {max}} = 160$ kg and $\text {BMI}_{\text {max}} = 52.8$ ${\textrm{kg m}}^-{1}$.

The subscript male or female indicates different PK parameter expressions depending on gender. The blood sampling site (arterial or venous) was available as a modeling covariate, but was automatically pruned by the symbolic regression algorithm.

The obtained model of Eq. 10 is less complex than the Eleveld model in [4], provided in Appendix B for reference, and comparable to simpler covariate models for propofol, such as [3, 16].

**Fig. 4:**
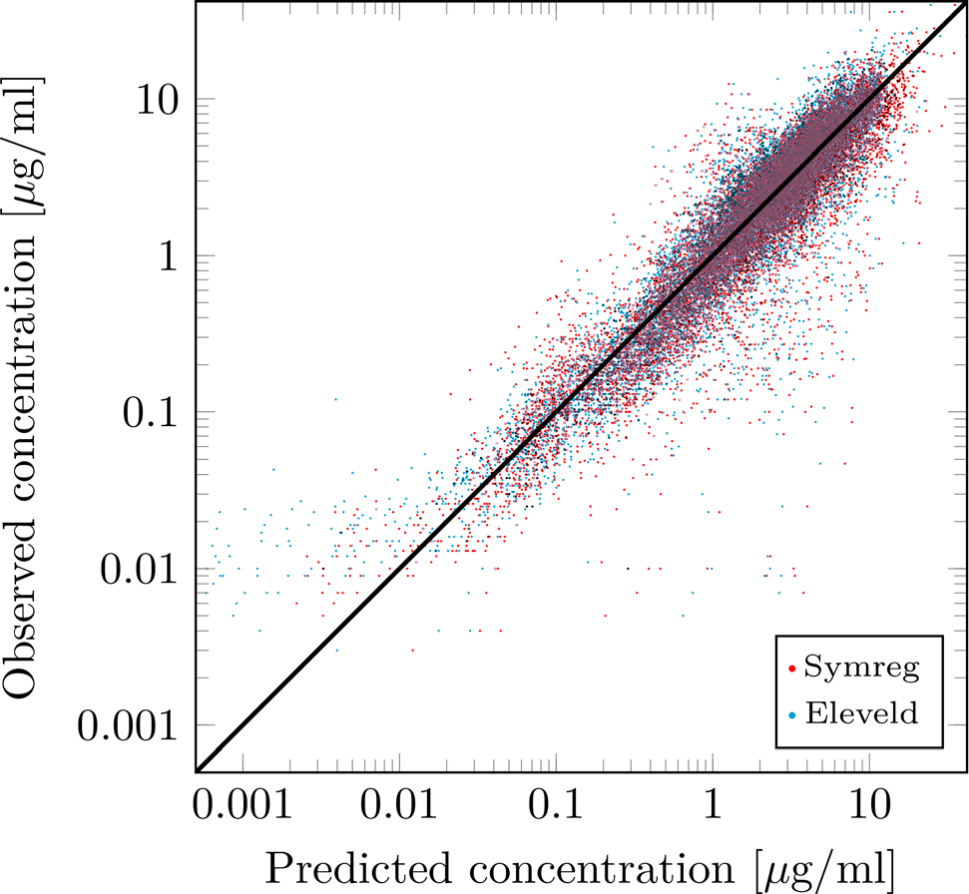
Predicted versus observed propofol concentrations of our covariate model (Symreg, red) compared to the Eleveld covariate model in [4] (Eleveld, blue) in logarithmic scale. The identity function, representing a perfect model fit, is shown in black

**Fig. 5:**
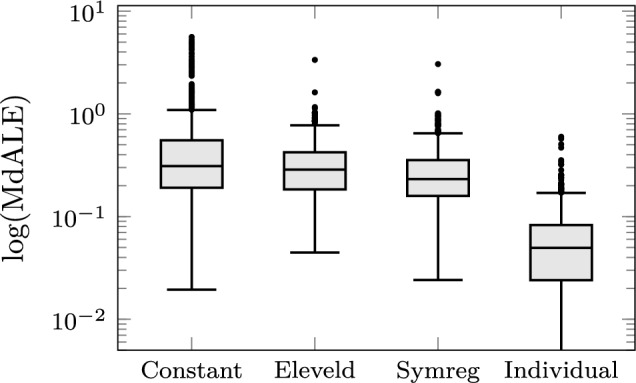
Comparison of prediction error MdALE Eq. 5 between predicted and observed propofol concentrations for pharmacokinetic models. Our covariate model is denoted Symreg and the Eleveld covariate model is described in [4]. The constant model represents one parameter set over the population and individual represents individual set of model parameters. The lower whisker for the individual models goes to zero

The predicted concentrations of the final covariate model on the Eleveld data set are shown in Fig. 4 together with the corresponding Eleveld model predictions. The distribution of errors between predicted and observed predictions is shown in the boxplot in Fig. 5, indicating comparable predictive capability of the models, despite our model being less complex, and involving fewer of the available covariates. For ease of comparison, we present the corresponding average prediction errors in Table 2. We compare our model to the Eleveld model, to a covariate-free PK model where all individuals share the same parameters values, and to covariate-free individual models with unique parameter sets. As expected, adding covariates explains some, but not all, variability between patients. This can be seen by comparing our model to the constant PK model and the individual models. As seen in Table 2, all of the prediction errors: MdLE, MdALE, MdPE, and MdAPE fall within clinically acceptable ranges, as presented further above.

**Table 2:** Comparison of prediction errors for the propofol data set in [4] (1,031 individuals) for several pharmacokinetic models, all trained with MdALE Eq. 5 as loss. Individual and constant PK model(s) are shown for comparison, representing best and worst case limits, respectively

Method	Mean MdALE	Mean MdLE	Mean MdAPE	Mean MdPE
Constant model	0.501	0.140	211	181
Eleveld model	0.325	0.0791	34.8	14.4
Symbolic regression	0.279	$-$0.0489	27.4	$-$0.266
Individual models	0.0623	1.65 $\cdot 10^{-4}$	6.21	0.0178

## Discussion

We have introduced a novel symbolic regression methodology for simultaneously automating the search for a suitable covariate model structure and optimization of its parameters. Similar to contemporary methodologies, it relies on a user-specified set of base expressions from which the covariant model can be composed. However, and in important contrast to contemporary methods, the need for a combinatorial search across combinations of these expressions is voided.

Throughout the paper, a propofol PK modeling example has been used as a demonstrator. Within this example, the proposed methodology manages to accomplish slightly better data fit than the result of state-of-the-art modeling [4] (see Figs. 4 and 5, Table 2), while relying on fewer covariates, see Eq. 10. The obtained values of individual volumes and clearances were comparable to those in [4].

The introduced methodology is broadly applicable to PK modeling from time series data, and likewise to pharmacodynamic (PD) modeling, and combined pharmacokinetic and pharmacodynamic (PK/PD) modeling. It’s main benefit lies in that it poses the search for a suitable PK model as symbolic regression with a smooth loss function. This, in combination with efficient methods for simulation and gradient computations [13] enables efficient model learning using back-propagation.

Another advantage is that the method can find covariate functions that are both simple and explain available data, while having a structure that would generally not be considered in a manual model structure search, unless explicitly supported by prior knowledge. However, the obtained covariate model is deterministic in the sense that we do not obtain a distribution over individual PK parameter values. It would be possible to integrate this methodology into the traditional mixed-effect modeling framework. Yet, this would be computationally much more demanding, which is why we propose first applying symbolic regression in a deterministic setting to arrive at a covariate model structure, and then (if desired) apply mixed-effect modeling to maximize parameter likelihood (with respect to some parameter priors and subject to the considered data) within the found structure.

In this paper, we have focused on models that are of sufficiently low complexity to be human-readable, which is achieved by enforcing sparsity of the neural network that constitutes the expression tree of the covariate model. If human readability is not necessary, an ordinary deep ANN could be employed instead. However, there is also a trade-off between fit to training data (expressiveness) and generalization to yet unseen data due to possible over-fitting. Enforcing human-readability naturally limits flexibility of the model, thus decreasing this risk of over-fitting. The ability to manually specify base expression also provides a means to integrate expert knowledge into the model. For example, a suspicion that a compartment volume should correlate to the square of patient age would motivate multiplication as a base expression. This enables incorporating expert knowledge into the model, for example a clearance to the power of 0.75 as in [32], or compartmental allometry as in [33].

We assessed the model’s out-of-sample prediction performance using a five-fold cross-validation. The data set was divided into five equal parts, and a covariate model was trained on four of the partitions, excluding one each time. The excluded partition, referred to as the validation set, was used to evaluate the model. This process was repeated for all five partitions to obtain an average predictive performance. The resulting mean (range) MdALE from cross-validation on the test sets was 0.303 (0.152-$-$0.423), similar to the mean MdALE of 0.279 in table 2. There was thus only a modest 10 % difference in mean error between the training and validation sets, which suggests no over-fitting issue. If such a problem had occurred, reducing the number of covariates and parameters (harder pruning) could have balanced the prediction errors on both sets.

The way we have employed the methodology here differs from how covariate models are usually trained using mixed-effect modeling. Rather than asserting parameter priors and selecting the most likely parameters from the posterior distribution that the data infers, we have chosen to train a scalar loss function, resulting in a deterministic covariate model. It would in theory be possible to embed our methodology within an inference engine, but at the cost of high computational cost. If a Bayesian interpretation is desired, a likely better alternative is to first run our methodology to arrive at a covariate model structure–as we have done in our example–and then assert parameter priors to the parameters of the resulting model, to finally apply mixed-effect modeling to compute the corresponding posteriors.

## Conclusion

We have presented a novel methodology for automatic and simultaneous covariate model structure discovery and parameter optimization. This model was demonstrated using an example on which it outperforms state-of-the-art modeling, in that it finds expressions that match data slightly better, while relying on notably fewer covariates. We conclude that the potential of automated model structure discovery is substantial; it could greatly optimize the process of pharmacometric covariate modeling. Additionally, it’s likely to provide an improved balance between model complexity and data fit. This improved balance is something that could be challenging to achieve when simply assessing a series of pre-set model structure candidates in sequence.

## Data Availability

Code and data for reproducing the results is available in [27].

## Appendix A. Hessian-based pruning

In this appendix we provide a mathematical interpretation of the roles played by the Hessian and parameter saliences in our pruning approach.

The effect of perturbing the parameter vector can be analyzed by approximating the loss function $J({\varvec{\gamma }})$ by a Taylor series. A perturbation $\partial {\varvec{\gamma }}$ of the parameter vector ${\varvec{\gamma }}$ will change the loss function by

$$\begin{aligned} \partial J({\varvec{\gamma }})=\, & {} \nabla J({\varvec{\gamma }}) \partial {\varvec{\gamma }}+ \frac{1}{2} \sum _i H_{ii} \partial {\gamma _i}^2 \\{} & {} + \frac{1}{2} \sum _{i \ne j} H_{ij} \partial \gamma _i \partial \gamma _j + {\mathcal {O}}(||\partial {\varvec{\gamma }}||^3), \end{aligned}$$

where $\partial \gamma _i$ are the components of $\partial {\varvec{\gamma }}$, $\nabla J({\varvec{\gamma }})$ is the gradient of the loss function and $H_{ij}$ are the elements of the Hessian matrix *H* of *J* with respect to ${\varvec{\gamma }}$ so that

$$\begin{aligned} \nabla J({\varvec{\gamma }}) = \frac{\partial J({\varvec{\gamma }})}{\partial {\varvec{\gamma }}}\, H_{ij} = \frac{\partial ^2 J({\varvec{\gamma }})}{\partial \gamma _i \partial \gamma _j}. \end{aligned}$$

The goal is to prune the parameters that are *least* sensitive, i.e. those that affect the loss *J* the least when perturbed. Even for our size of network, repeatedly computing the full Hessian *H* would notably slow down training of the symbolic regression model. Instead, we make a diagonal approximation of the Hessian, neglecting cross terms $H_{ij}$ with $i\ne j$.

Allowing training to converge before each pruning iteration, ensures that $\nabla J({\varvec{\gamma }}) = 0$ (or in practice negligible), thus enabling approximation of $\partial J({\varvec{\gamma }})$ by

$$\begin{aligned} \partial J({\varvec{\gamma }}) \approx \frac{1}{2} \sum _i H_{ii} \partial \gamma _i^2. \end{aligned}$$

This approximation is then used to form an importance measure (salience) of our network parameters, according to [28], where the salience of parameter $\gamma _i$ becomes

$$\begin{aligned} S(\gamma _i) = H_{ii} \gamma _i^2. \end{aligned}$$

## Appendix B. The Eleveld model

The Eleveld PK covariate model in [4] is given here,

$$\begin{aligned}&f_{\text {ageing}}(x) = \exp \left( x(\text {AGE}- \text {AGE}_{\text {ref}} ) \right) \\&f_{\text {sigmoid}}(x,E50,\lambda ) = \dfrac{x^\lambda }{x^\lambda + E50^\lambda } \\&f_{\text {central}}(x) = f_{\text {sigmoid}}(x,\theta _{12},1)\\&f_{\text {CLmaturation}} = f_{\text {sigmoid}}(\text {PMA},\theta _8,\theta _9)\\&f_{\text {Q3maturation}} = f_{\text {sigmoid}}(\text {AGE}+ 40\text {weeks},\theta _{14},1)\\&f_{\text {opioids}}(x) = {\left\{ \begin{array}{ll} 1, &{} \text {absence of opiates}\\ \exp \left( x \cdot \text {AGE}\right) , &{} \text {presence of opiates} \end{array}\right. }\\&f_{\text {Al-Sallami}} = {\left\{ \begin{array}{ll} \left( 0.88 + \frac{0.12}{1+\left( \text {AGE}/13.4 \right) ^{-12.7}} \right) \left( \frac{9270\cdot \text {WGT}}{6680 + 216 \text {BMI}}\right) , &{} \text {males}\\ \left( 1.11 + \frac{-0.89}{1+\left( \text {AGE}/7.1 \right) ^{-1.1}} \right) \left( \frac{9270\cdot \text {WGT}}{8780 + 244 \text {BMI}}\right) , &{} \text {females}\\ \end{array}\right. }\\&V_{1,\text {arterial}}(\text {L}) = \theta _1 \dfrac{f_\text {central}(\text {WGT})}{f_\text {central}(\text {WGT}_{\text {ref}})}\\&V_{1,\text {venous}}(\text {L}) = V_{1,\text {arterial}} \left( 1 + \theta _{17}(1 - f_\text {central}(\text {WGT}))\right) \\&V_2(\text {L}) = \theta _2 \dfrac{\text {WGT}}{\text {WGT}_{\text {ref}}} f_{\text {ageing}}(\theta _{10})\\&V_3(\text {L}) = \theta _3 \dfrac{f_\text {Al-Sallami}}{f_\text {Al-Sallami, ref}} f_\text {opioids}(\theta _{13})\\&CL(\text {L \ min}) = {\left\{ \begin{array}{ll} \theta _{4}, &{} \text {male}\\ \theta _{14}, &{} \text {female} \end{array}\right. }\Biggr \} \left( \dfrac{\text {WGT}}{\text {WGT}_{\text {ref}}}\right) ^{0.75}\\&\dfrac{f_\text {CLmaturation}}{f_\text {CLmaturation, ref}} f_\text {opioids}(\theta _{11})\\&Q_{2,\text {arterial}}(\mathrm{L \ min}) = \theta _5 \left( V_2/V_{2,\text {ref}} \right) ^{0.75} (1+ \theta _{16} ( 1 - f_\text {Q3maturation}))\\&Q_{2,\text {venous}}(\mathrm{L \ min}) = Q_{2,\text {arterial}} \cdot \theta _{18}\\&Q_3(\mathrm{L \ min}) = \theta _6 \left( V_3 /V_{3,\text {ref}} \right) ^{0.75} \dfrac{f_\text {Q3maturation}}{f_\text {Q3maturation, ref}} \end{aligned}$$

where the reference patient is male, 35 years old, 70 kg and 1.7 metres tall. All parameters values $\theta$ can be found in the original publication of [4].

## Footnotes

1 The original data set in [4] comprises of 1033 individuals but two of them were excluded due to lack of observation data.
